# The impact of national and international guidelines on newborn care in the nurseries of Piedmont and Aosta Valley, Italy

**DOI:** 10.1186/1471-2431-5-45

**Published:** 2005-12-05

**Authors:** Andrea Guala, Roberta Guarino, Mauro Zaffaroni, Claudio Martano, Claudio Fabris, Guido Pastore, Gianni Bona, the Neonatal Piedmont Group

**Affiliations:** 1 S.O.C. di Pediatria, Ospedale SS Pietro e Paolo, ASL 11, Borgosesia, Italy; 2 Clinica Pediatrica, Dipartimento di Scienze Mediche, Università del Piemonte Orientale, Novara, Italy; 3 Cattedra di Neonatologia, Dipartimento Materno-Infantile, Università di Torino, Torino, Italy

## Abstract

**Background:**

Care procedures for preventing neonatal diseases are carried out according to nurseries' traditions and may be not consistent with the evidence based medicine issues.

**Methods:**

A multi-centric survey was conducted in 2 Regions located in NW Italy (Piedmont and Aosta Valley) in order to collect information on some healthy newborn care procedures. During 2001, a questionnaire was sent to the chief pediatrician in charge to the all 33 nurseries of the region asking the methods used during 2000 as prevention of ophthalmia neonatorum, early and late hemorrhagic disease of newborn, umbilical cord care and recommendations of vitamin D administration. Thereafter, during 2004 the same questionnaire was sent to the 34 chief pediatrician of nurseries to evaluate if the procedures were changed during 2003 according to guidelines. The nurseries care for 32,516 newborns in 2000 and 37,414 in 2003.

**Results:**

Aminoglycoside eyes drops as prevention of ophthalmia neonatorum were the first choice in both periods (23 out 33 nurseries in 2000 and 24 out 34 in 2003 p > 0.05; the corresponding figures for newborns were18,984 out 32,516 newborns vs. 28,180 out of 37,414 p < 0.05). The umbilical cord care was carried out with alcohol in 12/33 centers (13,248 newborns) and dry gauze in 3/33 centers (2,130 newborns) in 2000, the corresponding figures in 2003 were 6/34 centers (p > 0.05), (6,380 newborns, p < 0.05) and 12/34 centers (p < 0.05), (18,123 newborns, p < 0.05). The percentage of newborns receiving of i.m. vitamin K. at birth increased during the study period (15,923/32,104 in 2000 vs. 19,684/37,414 in 2003, p < 0.01), but not the number of nurseries (16 in 2000 and 17 in 2003 p > 0.05). The numbers of parents of newborns who receive the recommendations of oral vitamin K during the first months life decreased from 2000 (25,516/30,606) to 2003 (29,808/37,414, p < 0.01) as well as for Vitamin D recommendation (14,582/30,616 in 2000 vs. 11,051/37,414 in 2003, p < 0.01). Oral vitamin K during the first months of life was recommended by 25 nurseries in 2000 and 27 in 2003 (p > 0.05), the corresponding figures for Vitamin D were 15 and 14 (p > 0.05).

**Conclusion:**

In the present study a large variability of procedures among the nurseries was observed. During the study periods, guidelines and evidence based medicine issues have only partially modified the neonatal care procedures In Piedmont and Aosta Valley nurseries. These observations suggest to implement local forum/consensus conference to standardized procedures as much as possible.

## Background

Immediately after delivery healthy newborn underwent care procedures to prevent some diseases. Usually these interventions are done according to nurseries' common place and traditions which sometimes are not consistent with established guidelines and/or with evidence based medicine issues.

The key aims of this descriptive multi-centric survey are: 1) to compare the procedures applied to during 2000 and during 2003 in Piedmont and Aosta Valley nurseries for the prevention of ophthalmia neonatorum, of early and late hemorrhagic disease of newborn (HDN) through the administration of vitamin K, of rickets through the recommendation of vitamin D and for the care of the umbilical cord and 2) to evaluate the impact of Italian guidelines [14], when available, and/or international ones [1,2,5,18] on the newborns care.

## Methods

In 2001 and 2004 an identical questionnaire (see Additional file 1) was sent to the chief pediatrician and ward nurse of all nurseries located in 2 Regions of North West Italy (Piedmont and Aosta Valley) in order to collect information on procedures used to prevent the ophthalmia neonatorum, the early and late HDN, the rickets and methods employed for the umbilical cord care and, in addition, the number of newborns born in each hospital in 2000 and in 2003. The forms, composed of 10 closed items and 9 open ones, were filled by the pediatrician in charge.

In 2000, there were 33 nurseries operating (1 was temporary closed), whereas in 2003 there were 34 active nurseries in Piedmont and Aosta Valley Regions. The number of newborns born in each hospital ranged from 400/year to 4000/year. For these reason the "weight" of each nurseries included in the present study is very different. In addition, in Italy, the policy is that there is just one way of doing things in each hospital, mandated by the chief pediatrician. For these reason we used as unit of analysis both the number of the nurseries and the number of newborns.

The differences among the distributions of nurseries who used different approach and of newborns who received different procedures were statistically analyzed using chi-square test.

## Results

During 2000, in Piedmont and Aosta Valley 32,516 newborns were born in the 33 nurseries. For 2003, the corresponding figures were 37,414 newborns born in the 34 nurseries. About 25% of newborns were cared in 2 nurseries operating in Turin, the largest city of both Regions.

All but one colleagues answered questions on the prevention of ophthalmia neonatorum in 2000, while all questionnaires were filled in 2003. The majority of the nurseries used amino glycoside as prophylaxis (23 in 2000 and 24 in 2003 p > 0.05; caring on 18,984 and 28,180 newborns respectively, p < 0.05). Over 5400 newborns (16%) received chloramphenicol eye-drops in 2000 and in the second period over 6700 (18%) (p < 0.05, table 1).

**Table 1:** Ophthalmia neonatorum prophylaxis: drugs used in the nurseries of Piedmont and Aosta Valley, during 2000 and 2003

**Year of survey**	2000			2003		
**Drug**	**N. nurseries**	**N. newborns**	**%**	**N. nurseries**	**N. newborns**	**%**

Tobramycin	12	15,168	47.2	17	20,879	55.8
Cloramphenicol	7	5,457	17.0	8	6,720	18.0
Gentamicin	6	3,816	11.8	7	7,301	19.5
Clortetraciclyne	3	3,614	11.1	-	-	-
Other	4	4,129	12.9	2	2,514	6.7
Total	32	32,104	100.0	34	37,414	100.0
Data not available	1	412	1.3			

Table 2 shown the distribution of procedures used for the umbilical cord care. In 2000, the umbilical cord care was done with alcohol in 12 nurseries (who cared 13,248 newborns, 41%), in 4 nurseries (6,265 newborns, 19%) with micronized silver spray, in 4 nurseries (1,681 of newborns, 5%) with eosine and only in 3 nurseries (2,130 newborns, 7%) with dry gauze. In 2003, 12 nurseries (18,123 of newborns, 48 %) used for cord stump care dry gauze (comparing the number of newborns 2000 vs 2003, p < 0.05), 7 nurseries (5,613 newborns,15 %) arnica/echinacea and only 6 nurseries (6380 newborns,17 %) alcohol. The number of centers who used dry gauze or arnica/echinacea increased from 2000 to 2003 (p < 0.05), whereas the number of centers who used alcohol decreased (p < 0.05).

**Table 2:** Umbilical cord care used in the nurseries of Piedmont and Aosta Valley, during 2000 and 2003

**Year of survey**	2000			2003		
**Methods**	**N. nurseries**	**N. newborns**	**%**	**N. nurseries**	**N. newborns**	**%**

Alcohol	12	13,248	40.6	6	6,380	17.0
Silver micronized	4	6,265	19.2	2	1,364	3.6
Eosine 2%	4	1,681	5.2	2	990	2.6
Arnica/echinacea	3	3,221	9.9	7	5,613	15.0
Clorexidine	3	2,313	7.1	1	1,197	3.3
Dry gauze	3	2,130	6.5	12	18,123	48.5
Peroxid water	2	1,357	4.2	3	2,559	6.8
Salicylic sugar	1	1,216	3.7	-	-	-
Triple dye	1	1,165	3.6	1	1,188	3.2
Total	33	32,516	100.0	34	37,414	100.0

All colleagues but one replied on early HDN prophylaxis used in 2000, while all replied in 2003 (table 3). In 2000, vitamin K was administered per os to 16,261 newborns (50%) cared in 17 nurseries. Among these 17 centers, 13 used intramuscolar (i.m.) vitamin K in pre-term newborns, in low birth weight babies and when there was evidence of fetal distress. In 2003, 17 nurseries used vitamin K per os (17,730 newborns, 47 %).

**Table 3:** Early hemorrhagic disease of newborn prophylaxis according to vitamin K schedule in the nurseries of Piedmont and Aosta Valley during 2000 and 2003

**Year of survey**	2000			2003		
**Administration**	**N. nurseries**	**N. newborns**	**%**	**N. nurseries**	**N. newborns**	**%**

Oral	17	16,261	50.6	17	17,730	47.4
Intramuscular	15	15,923	49.4	17	19,684	52.6
Total	32	32,104	100.0	34	37,414	100.0
Data not available	1	412	1.3			

Data on late HDN are shown in table 4. In 2000, 25 centers prescribed oral vitamin K for 3–6 months. Among the 15 nurseries, where vitamin K prophylaxis was done by i.m. after the birth, only 6 did not prescribe additional oral vitamin K dose. In 2003, 27 nurseries administered oral vitamin K. Among the 17 centers that used i.m. vitamin K after the birth, 7 did not suggest further oral doses. The number of newborn who received the recommendation of oral vitamin K increase from 25,516 during 2000 to 29,808 thereafter (p < 0.05), whereas the number of nurseries was not statistically different in the two periods.

**Table 4:** Late hemorrhagic disease of newborn: prophylaxis with oral vitamin K suggested by the nurseries of Piedmont and Aosta Valley, during 2000 and 2003

**Year of survey**	2000			2003		
**Oral vitamin K suggested**	**N. nurseries**	**N. newborns**	**%**	**N. nurseries**	**N. newborns**	**%**

Yes	25	25,516	83.4	27	29,808	79.7
No	6	5,170	16.6	7	7,606	20,3
Total	31	30,606	100.0	34	37,414	100.0
Data not available	2	1,910	5.9			

In 2000, at discharge the health care providers of 15 centers recommended vitamin D to all newborns (14,582 newborns, 48 %) (regardless pigmentation of skin and period of birth) for different period of time ranging between 3 months to 1 year (table 5). Among the 16 centers that did not recommend routinely the use of vitamin D, 10 suggested vitamin D at the discharge of pre-term or small for gestational age newborns, 2 suggested only to babies born in winter, 1 suggested only to dark-skinned newborns and 3 never. In 2003, 14 nurseries (11,051 newborns, 30 %) prescribed vitamin D to all newborns (comparing the number of newborns 2000 vs. 2003, p < 0.05), whereas the number of nurseries was not statistically different in the two periods. Among centers in 2003, that did not routinely suggest vitamin D, 11 prescribed it only to pre-term newborns, 4 only to dark-skinned babies and 5 never.

**Table 5:** Rickets prophylaxis: vitamin D suggested at discharge in the nurseries of Piedmont and Aosta Valley during 2000 and 2003

**Year of survey**	2000			2003		
**Oral vitamin D suggested**	**N. nurseries**	**N. newborns**	**%**	**N. nurseries**	**N. newborns**	**%**

yes	15	14,582	47.6	14	11,051	29.5
no	16	16,104	52.4	20	26,363	70.5
Total	31	30,606	100.0	34	37,414	100.0
Data not available	2	1,910	5.9			

## Discussion

Clinical guidelines provide specific recommendations for practice. Various organization developed systematic programs to produce consensus statements and standards for good medical care. Under the movement of evidence based medicine, systematic literature review and linkage of recommendations to supporting evidence became essential. Nevertheless, despite the availability of the same body of evidence (MEDLINE and The Cochrane Library) recommendations differ and the translation in good practice is not linear [4,12]. Our study reports an heterogeneity of procedures in the nurseries of 2 Regions located in NW of Italy (Piedmont and Aosta Valley) to prevent potentially severe diseases in healthy newborns. In every nursery the prophylactic interventions are mainly related to long lasting habit/commonplace and previously developed experiences. During the study period, many guidelines or EBM reviews on the newborns care have been released both from Italian Health Care Society both from international Society. In addition a variety of published papers, meta-analysis reports, recommendations developed by ad-hoc committees are available, for each of the four prophylactic interventions described in the present study [1,2,5,6,14,15,17,18].

According to recommendations of the Canadian Society of Pediatrics [5] and to the guidelines issued by the WHO [15], the choice of antibiotic for preventing ophthalmia neonatorum should be related to local epidemiology of the most frequent bacteria.

Guidelines have been developed by the WHO [17] and by Cochrane Library [18] for the umbilical cord care and an Italian evidence based study was recently published [11]. Delay in the cord stump detachment might cause parents' anxiety [3] and different procedures to cord care have been compared [8,13].

A lot of studies and reviews are available on the prevention of early HND: the i.m. via is effective as the oral administration without severe side-effects [7]. However the low compliance of the oral administration did can not completely exclude the late HND [9].

Recommendations of the American Society of Pediatrics are available on vitamin D administration to prevent rickets [2]. The prevention might begin during pregnancy [6]. In Piedmont, due to the immigration from Africa in recent years, rickets is diagnosed almost exclusively in dark skinned children (personal data) and the prescription of vitamin D during early after birth is not mandatory in non dark skinned infants.

During 2000, in the large majority of nurseries the used procedures differ from the guidelines developed by the Italian scientific Society. Many scientific literature review and recommendations from several pediatric academies have been published after 2000 and this might justify the variability of conduct observed during the first period of the our survey. In the 2003 (second period of the present study) the results showed a more strict adherence to the available guidelines. The 2 largest nurseries, which care about one quarter of newborns born each year in Piedmont, changed the procedures according to evidence of available reviews. While the medium size (1000–2000 newborns/year) or small size (less than 1000 newborns/year) nurseries show a more slow trend to adapt their care standard according to guidelines. In any case, the health provider conduct showed a large variability even if Italian guidelines, implemented by the scientific societies, were published [14].

Besides, medical prescription or diagnostic/therapeutic pathway are not exclusively tied to scientific evidences. Drug prescriptions are not only a medical act coming from diagnosis evidence, but are often strongly influenced by psychological, cultural and socio-economic elements, as recently confirmed in Italy by APE (Attitudini Prescrittive in Pediatria) study [10,16].

## Conclusion

It is unclear why clinician fail to follow the guidelines. Although the quality of evidence-based guidelines in pediatrics is high [19], it is always noticed the gap between the scientific evidence and the update and the application of that evidence in practice [20]. Reliance on guidelines may prevent the development of serious co-morbidities. The large variability of preventive intervention observed in our survey might be an opportunity for implementing a forum among the pediatricians and the neonatologists to developing or reinforce evidence-based guidelines. Health care organization should increase their educational efforts. Actually, during 2005 and 2006 a number of meetings are planned and supported by the regional piedmont section of the Italian Society of Pediatrics, by the Italian Society of Neonatology and by the Associazione Culturale Pediatri to achieve this goal.

## Competing interests

The author(s) declare that they have no competing interests.

## Authors' contributions

AG had the primary responsibility for protocol development and writing the manuscript. RG contributed to elaboration of data. MZ, CM, CF, GB contributed to the writing the manuscript. GP supervised the design and the execution of the study, and contributed to the writing the manuscript.

**The Neonatal Piedmont Group**: Besenzon L. (Saluzzo, Savigliano), Bonenti G. (Rivoli), Campra D. (Borgosesia), Castella E. (Tortona), Castelli G. (Ceva, Mondovì), Cattaneo G. (Moncalieri), Cattrini C. (Domodossola, Verbania), Cocuzza S. (Alba, Bra), Coscia A. (Torino), De Franco S. (Novara), De Marinis S. (Cuneo), Favetta S. (Chivasso), Frigerio M. (Torino), Galligani L. (Biella), Giaretto G. (Courgnè, Ivrea), Gomirato G. (Torino), Grazia G. (Pinerolo), Guala G. (Torino), Lancione F. (Chieri), Machado E. (Aosta), Maganuco S. (Ciriè, Venaria Reale), Papili F. (Casale Monferrato), Pesce F. (Alessandria), Provera S. (Vercelli), Rigardo S. (Acqui Terme, Novi Ligure), Roberi P. (Carmagnola), Rossi E. (Torino), Vitali M. (Borgomanero), Zannino L. (Asti).

## Pre-publication history

The pre-publication history for this paper can be accessed here:



## Supplementary Material

Additional File 1 the additional file nidi questionario.doc report the questionnaire used for the present study. Click here for file
