# Supplementary material for: The impact of national and international guidelines on newborn care in the nurseries of Piedmont and Aosta Valley, Italy
Source: BMC Pediatr. 2005 Dec 5;5:45. doi: 10.1186/1471-2431-5-45 (PMC1315318; doi:10.1186/1471-2431-5-45)
Supplement: Additional File 1 — the additional file nidi questionario.doc report the questionnaire used for the present study. [file 1471-2431-5-45-S1.doc]

*Novara, 21 marzo 2001*

*Gentile Primario e Gentile Caposala,*

*cogliamo l’occasione per informarvi dei risultati della indagine sulla posizione nel sonno al Nido dei neonati fisiologici effettuata lo scorso anno.*

*Allegato è il testo del lavoro che si propone per la pubblicazione su Rivista Italiana di Pediatria.*

*I risultati della ricerca verranno proposti a Savigliano al prossimo Convegno regionale di Pediatria e Neonatologia.*

*E’ in preparazione anche un lavoro per Neonatologica Infermieristica con il nome di tutte la Capo-sala che hanno collaborato allo studio.*

*Vi richiediamo ora ancora un piccolo sforzo nel collaborare a una nuova indagine sulle procedure assistenziali effettuate nei Nidi delle nostre Regioni. E’ stato elaborato il questionario allegato al fine di conoscere quali sono le procedure e le indicazioni per le profilassi alla nascita, la medicazione del moncone ombelicale e la prescrizione di vitamine alla dimissione.*

*Vi preghiamo, quindi, di restituire le schede compilate a:*

*Prof. Gianni Bona Clinica Pediatrica di Novara FAX 0321.3733.598*

*“Ospedale Maggiore”, C.so Mazzini, 18 - 28100 Novara .*

*Per ogni dubbio o domande è possibile rivolgersi a:*

*Dr. Andrea Guala tel. 0163 203111*

*Dr. Mauro Zaffaroni tel. 0321.3733670 E.mail: maurozaff@libero.it*

*Ringraziando per la collaborazione, porgiamo i migliori saluti.*

*Gianni Bona, Andrea Guala e Mauro Zaffaroni*

S.I.N. S.I.P.

## **Sezione Piemonte Sezione Piemonte**

## **e Val d’Aosta e Val d’Aosta**

Indagine conoscitiva sulle procedure assistenziali nei

Punti Nascita di Piemonte e Val d’Aosta

Reparto di Neonatologia Ospedale di ….………………..…………………………..

Primario …………………………… Caposala Sig.ra/re…………………………..

Numero neonati nel 2000 : ………………. Tel. …………………………..

**--------------------------------------------------------------------------------------------------------------**

- **VITAMINA K alla nascita**

La vitamina K viene somministrata ai neonati ? SI  NO 

Se sì, quando ? In sala parto  Subito al Nido  In 1° giornata 

Come ? Per os  i.m.  Quanto? in mg : ……………………

La via di somministrazione e la quantità variano a secondo del peso, dell’età gestazionale

del neonato, del tipo di parto ? (specificare): ………………………………………………

…………………………………………………………………………………….……………

- PROFILASSI OCULARE alla Credè

Il disinfettante oculare viene somministrato ai neonati? SI  NO 

Se sì, quando ? In sala parto  Subito al Nido  In 1° giornata 

Quale disinfettante viene usato ? (specificare) : ………………………………………………………………………………………………

Quale dose viene somministrata ? (specificare): ..……………………………………….

- MEDICAZIONE DEL MONCONE OMBELICALE:

Specificare la procedura ed il prodotto usato: ………………………………………….

………………………………………………………………………………………………

quante volte ? ………………………………………………………………………………

Si usano strumenti sterili per la recisione ? SI  NO 

- INDICAZIONI ALLA DIMISSIONE:

VITAMINA D

Alla dimissione del neonato viene consigliata la somministrazione di Vitamina D?

SI  NO  A volte 

Se sì, : quale prodotto ? …………………… quale dose ? ………………………

per quanto tempo ………………………………………………………………

**Se a volte, in quali casi ? (specificare): ………………………………………………**

……………………………………………………………………………………………

### VITAMINA K

Alla dimissione del neonato viene consigliata la somministrazione di vitamina K ?

**SI  NO  A volte **

**Se sì: quale prodotto ? ……………………… quale dose? ………………………**

**per quanto tempo ……………………………………………………………...**

**Se a volte, in quali casi ? (specificare): ………………………………………………**

**……………………………………………………………………………………………**

**Dear Director and Dear Ward Nurse,**

We have pleasure in taking the opportunity to inform you about the results of the research concerning the sleeping position of newborn babies at the hospital nursery, carried out last year. Please find herewith attached the whole text of the research, due to be published on the Italian Paediatrics Magazine.

The results of the research will be illustrated on the occasion of the next regional Convention of Paediatrics and Neonatology.

We are also pleased to inform you that a further study on Nursery Neonatology is being prepared, indicating the names of all Ward Nurses who participated in it.

We are however asking you a further little effort in co-operating with a new inquiry on the assistential procedures of the nurseries of our region.

The attached questionnaire has been prepared with the main purpose of gaining a deeper knowledge of the prophylaxis procedures followed and of the indications given at the baby birth, in taking care of his/her umbilical cord as well as in prescribing vitamins at his/her discharge from hospital.

You are then kindly requested to fill in the forms and send them back to Prof. Bona or Dr. Zaffaroni, Paediatric Clinic of University, Novara.

**For any doubts or queries you may have, please do not hesitate to contact Dr. Guala.**

**Thank you so much for your kind co-operation.**

**Kind regards**

S.I.N. S.I.P.

## **Sezione Piemonte Sezione Piemonte**

## **e Val d’Aosta e Val d’Aosta**

**COGNITIVE INQUIRY ON THE ASSISTENTIAL PROCEDURES IN THE NEONATOLOGY DIVISIONS OF PIEMONTE AND AOSTA VALLEY**

### Neonatology Department, Hospital of…........................

**Director….**

**Ward Nurse…**

**Number of newborn infants in … 2000/2003 ….**

**K VITAMIN at BIRTH.**

**Is K vitamin administered to the newborn? Yes No**

**If yes, when? In the delivery room Immediately in the nursery On the first day**

**How? Os Intramuscolar**

**How much? (mg) ….**

The administration method and the dose may vary, depending on the newborn weight and gestational age, as well as on the type of delivery? (specify)……..

**OCULAR PROPHYLAXIS (Credè method)**

**Is the ocular disinfectant administered to the newborn? Yes No**

**If yes, when? In delivery room Immediately in the nursery On the first day**

**Type of disinfectant used (specify) ……..**

**Dose of disinfectant administered (specify) ……**

**CARE OF THE UMBILICAL CORD**

**Please indicate the procedure followed along with the product employed…….**

**How often?............................................**

**Are sterilized instruments employed for cutting the cord?**

**D VITAMIN.**

**Is vitamin D routinely recommended at newborn discharge? Yes No Sometimes**

**If yes, what product?……. what dose?…… for how long? …..**

**If sometimes, in what cases (specify)? …….**

**K VITAMIN**

**Is vitamin K routinely recommended at newborn discharge? Yes No Sometimes**

**If yes, what product?……. what dose?…… for how long? …..**

**If sometimes, in what cases (specify)? …….**
